# Association between levonorgestrel emergency contraception and the risk of ectopic pregnancy: a multicenter case-control study

**DOI:** 10.1038/srep08487

**Published:** 2015-02-12

**Authors:** Jian Zhang, Cheng Li, Wei-Hong Zhao, Xiaowei Xi, Shu-Jun Cao, Hua Ping, Guo-Juan Qin, Linan Cheng, He-Feng Huang

**Affiliations:** 1 Department of Obstetrics and Gynecology, International Peace Maternity and Child Health Hospital, School of Medicine, Shanghai Jiaotong University, Shanghai 200030, China; 2 Department of Obstetrics and Gynecology, Shanghai First People's Hospital, Shanghai Jiaotong University, Shanghai 200080, China; 3 Department of Obstetrics and Gynecology, Songjiang Central Hospital, Shanghai 201600, China; 4 Department of Obstetrics and Gynecology, Songjiang Maternity and Child Health Hospital, Shanghai 201620, China; 5 Department of Obstetrics and Gynecology, Minhang Central Hospital, Shanghai 201100, China; 6 Shanghai Institute of Planned Parenthood Research, Shanghai 200030, China; 7 Department of Reproductive Medicine, International Peace Maternity and Child Health Hospital, School of Medicine, Shanghai Jiaotong University, Shanghai 200030, China

## Abstract

Cases of ectopic pregnancy (EP) following levonorgestrel emergency contraception (LNG-EC) failure have been reported continuously, but whether there is an association between EP risk and LNG-EC is unclear. We concluded a case-control study to explore this association by recruiting 2,411 EP patients as case group, and 2,416 women with intrauterine pregnancy and 2,419 non-pregnant women as control groups. Odds ratios (ORs) and their 95% confidential intervals (CIs) were calculated and adjusted for potential confounding factors. Previous use of LNG-EC was not correlated with the EP. Compared to women who did not use contraceptives, current use of LNG-EC reduced the risk for intrauterine pregnancy (Adjusted OR [AOR] = 0.20, 95%CI: 0.14–0.27), but did not increase the risk for EP (AOR_2_ = 1.04, 95%CI: 0.76–1.42). Furthermore, compared to women who did not have further act of intercourse, women with unprotected further act of intercourse were at a higher risk of EP (AOR_1_ = 2.35, 95%CI: 1.17–4.71), and women with repeated use of LNG-EC for further intercourse during the same cycle was also associated with a higher risk for EP (AOR_1_ = 3.08, 95%CI: 1.09–8.71; AOR_2_ = 2.49, 95%CI: 1.00–6.19). A better understanding of the risk of EP following LNG-EC failure can optimize LNG-EC use and thus reduce the risk of EP.

Ectopic pregnancy (EP) accounts for 1.3–2.0% of all naturally conceived pregnancies^1^,^2^; it remains the leading cause of maternal mortality in the first trimester of pregnancy and accounts for almost 5% of maternal deaths^3^. Over the past decades, the incidence of EP has increased as a result of an increased and persistent exposure to its risk factors^4^. The popularity of contraceptive methods that predispose users to EP following contraceptive failure also contributes to the increased incidence of EP^5^. Any form of contraception is associated with a decrease in the number of EPs it reduces the chances of pregnancy; however, in the case of contraceptive failure, the risk of EP varies across the different contraceptive methods^6^. Thorburn *et al* showed that condom use did not increase the risk of EP^7^. Further, some meta-analyses found that oral contraceptive pills (OCPs), intrauterine devices (IUDs), and female sterilization could increase the risk of EP to different degrees in cases of contraceptive failure^6^,^8^.

Emergency contraception pills (ECPs) are currently widely accepted and used by women after unprotected intercourse to prevent unwanted pregnancies. Levonorgestrel-only pills for emergency contraception (LNG-EC) are available in the over-the-counter form in many countries including China, and they can prevent unwanted pregnancies with an efficacy of 52–94% when used within 120 h of unprotected intercourse^9^,^10^,^11^,^12^,^13^. Like other contraceptive methods, LNG-EC reduces the chance of pregnancy, including both intrauterine and occasional ectopic pregnancy; however, cases of EP following LNG-EC failure have been reported by the present author and other researchers in various countries^14^,^15^,^16^,^17^. The New Zealand Medicines and Medical Devices Safety Authority warned ECP users that in the cases of contraception failure, “the possibility of EP should be considered if the pregnancy test is positive”^18^; the same concern was also raised in other countries including the USA^19^. Cleland *et al.* conducted a systematic review by including studies published up to August 2009, and drew the conclusion that the incidence of EP following LNG-EC failure was 1.6% which did not exceed the incidence in general female population. However, most clinical trials, including in this systemic review, set contraception failure and becoming pregnancy as the primary endpoint, and did not report whether pregnancies following LNG-EC failure were intrauterine or extra-uterine. Cleland *et al.* obtained the information for their study by contacting the authors of the other studies; this methods seems unreliable, and therefore their findings require further study^20^. Studies in other countries have reported a higher incidence. For example, a study conducted in Hong Kong reported that 128 out of 10,845 women using LNG-EC got with pregnant, and three of them had EP, which accounts for an EP incidence of 2.3% following LNG-EC failure^21^. Another study in France reported a higher incidence (3/73, 4.1%) of EP following LNG-EC failure^22^.

As a consequence of the over-the-counter availability of LNG-EC, the number of LNG-EC users has gone up rapidly in the last several years. The two largest companies manufacturing LNG-EC pills in China announced that in 2012 around 790,000 boxes containing either two 1.5 mg pills or four 0.75 mg pills were sold in the city of Shanghai alone (Cheng, personal data). In the course of our clinical practice too, we have noticed that more and more Chinese women with EP report taking LNG-EC during their conception cycle. Given this, there is a clear need to examine the EP incidence associated with LNG-EC failure among Chinese women. This study was conducted with the aim of understanding the risk of EP associated with LNG-EC use in the Chinese population. Prospective studies are difficult since women have easy access to LNG-EC without a doctor's prescription, therefore, we conducted this retrospective case-control study at five hospitals in Shanghai, China.

## Results

**Figure 1:**
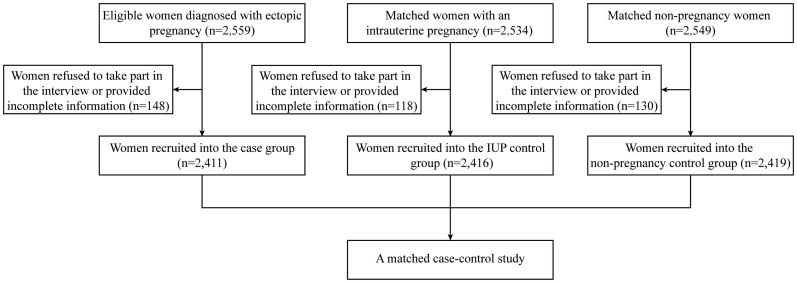
Recruitment profile of this study.

After excluding 148 EP patients, 118 intrauterine pregnancy (IUP) women and 130 non-pregnant women who refused to take part in the interview and withdrew from the study or provided incomplete information, a final of 2411 women in the EP group, and 2416 matched women in the IUP group and 2419 matched women in the none-pregnant group were included in analysis, with a response rate of 94.18% (recruitment profile shown in Figure 1).

The differences in socio-demographic characteristics, history of reproduction, gynecological disease, previous surgery, and previous contraceptive use in the women in these three groups are summarized in Table 1. No significant differences were found in age, marital status, or hospitals among these three groups. The proportions of women born outside of Shanghai, self-employed or unemployed, and with lower education was significantly greater in the EP group than in the IUP and non-pregnant groups. Furthermore, the proportion of parous women or women with a previous EP, previous *Chlamydia trachomatis* (CT) infection, history of infertility, previous adnexal surgery or appendectomy was significantly greater in the EP group than in the IUP and non-pregnant group. Compared to women in the IUP and non-pregnant group, women with EP were more likely to have previously used IUDs, and less likely to have had previous experience using a condom or other contraceptives.

### Association between previous use of LNG-EC and EP risk

Table 2 presents the results of the analyses on the association between previous use of LNG-EC and EP risk (women who had no previous experience in LNG-EC use were used as a reference). Multivariate analysis revealed that previous use of LNG-EC was not correlated with the risk of EP (AOR_1_ = 0.99, 95% CI: 0.85–1.18; AOR_2_ = 0.95, 95% CI: 0.82–1.10); the number of times LNG-EC had been used previously also did not have any significant effect on EP occurrence (P_1_ for trend = 0.71, P_2_ for trend = 0.67). After adjustment, the tests for trends did not reveal any significant association between the number of times LNG-EC was used within the last one year to the current cycle and the risk of EP (P_1_ for trend = 0.47, P_2_ for trend = 0.75). Moreover, there was no significant association between the risk of EP and the reduction in the time interval between the last LNG-EC use and the current cycle (P_1_ for trend = 0.37, P_2_ for trend = 0.86).

### Association between current use of LNG-EC and EP risk

The results of the risk analyses for EP following LNG-EC use in the current cycle are shown in Table 3. Univariate analysis found that compared to non-users of contraceptives, current users of LNG-EC had a reduced risk of both intrauterine pregnancies (OR for IUP = 0.26, 95% CI: 0.19–0.35, not shown in Table 3) and EP (OR_1_ = 0.72, 95% CI 0.56–0.94), but an increased risk for EP in the case of contraceptive failure (OR_2_ = 2.79, 95% CI: 2.27–3.43). After adjustment for confounding factors, the adjusted OR for IUP was 0.20 (95% CI: 0.14–0.27, not shown in Table 3), and the adjusted OR_1_ slightly changed to 1.04 (95% CI: 0.76–1.42), but the adjusted OR_2_ went up to 5.29 (95% CI: 4.07–6.87). Multivariate analysis indicated that in the case of LNG-EC failure, the risk of EP did not increased as the delay of taking LNG-EC after having unprotected intercourse (P_2_ for trend = 0.74). The time interval between last menstrual period (LMP) and current use of LNG-EC seemed to correlate with the risk of EP following LNG-EC failure. According to the results of multivariate analysis, the AOR_2_ was 4.57 (95% CI: 2.82–7.42) when LNG-EC was taken within 12 days after LMP; it was 4.28 (95% CI: 2.64–6.95) when taken within 13–14 days; it was 3.91 (95% CI: 2.14–7.14) when taken within 15–16 days; and it was 5.65 (95% CI: 3.86–8.27) when taken more than 17 days after LMP. There was also an association between the LNG-EC regime and risk of EP in the case of EC failure. The AOR_2_ was 5.15 (95% CI: 3.87–6.86) in women who took a single dose of 1.5 mg, which was higher than that in women who took two doses of 0.75 mg with 12 apart (AOR_2_ = 3.14, 95% CI: 1.73–5.71). In addition, we also found that among women who took LNG-EC in the current cycle, those who engaged in further acts of intercourse after taking LNG-EC had no significant risk of EP in the case of EC failure when compared to those who did not have further act of intercourse (AOR_2_ = 1.15, 95% CI: 0.74–1.79). Furthermore, compared to women who did not engage in further act of intercourse, women without using any contraceptive methods for the further act of intercourse were at a higher risk of EP (AOR_1_ = 2.35, 95% CI: 1.17–4.71), and women with repeated use of LNG-EC for further intercourse during the same cycle was also associated with a higher risk for EP (AOR_1_ = 3.08, 95% CI: 1.09–8.71; AOR_2_ = 2.49, 95% CI: 1.00–6.19).

## Discussion

In this study, we explored the association between EP risk and previous or current use of LNG-EC, as this has not been systematically studied in the past. Our results showed that LNG-EC, like other contraceptive methods, could reduce the incidence of unwanted pregnancies including EP. Despite the low pregnancy rate following the use of LNG-EC, cases of EP following LNG-EC failure have still been reported^14^,^15^,^16^,^17^. There could be three major reasons for this: (1) With a failure rate of 0.2–3.3%, LNG-EC is less effective in preventing pregnancies than other contraceptive methods such as OCPs and IUDs^23^. (2) More and more Chinese women are aware of and use LNG-EC because of its easy accessibility (over-the-counter availability since 1998) and wide marketing. (3) Although there is better awareness on how to use LNG-EC correctly in recent years, there are still some women who use LNG-EC improperly without referring to the manufacturer's instructions strictly.

Some traditional risk factors for EP, like parity, previous EP, CT infection, previous infertility, previous adnexal surgery, and previous use of IUDs, have been demonstrated in the literatures^24^,^25^. From this study, these factors also showed significant differences among three groups, which was identical to the previous studies. In order to clearly observe the association between the EP risk and the use of LNG-EC, these factors were treated as confounding factors, and included into the adjustment models.

To our knowledge, this is the first study analyzing the association between previous use of LNG-EC and the current pregnancy outcome. Our data do not show a positive association between previous use of LNG-EC and risk of EP or IUP, and no dose-effect response was observed either. This is mainly because the half-life of LNG-EC is about 24 h *in vivo*, and the plasma concentration of LNG-EC returns to that of the pre-administration level in about five days^26^. This also explains why there was no influence of the time interval between last use of LNG-EC and the current cycle of conception or menstruation on the pregnancy outcome. Thus, it seems that repeated use of LNG-EC in the previous cycle rather than just previous use does not carry the risk of EP.

The use of LNG-EC in the current cycle could reduce the risk of IUP without increasing the risk for EP. However, in the case of EC failure, LNG-EC users were at a significantly higher risk of having an EP when compared to non-users of contraceptives. Studies have found that a high dose of progesterone could affect the function of the human fallopian tube by reducing the activity of tubal cilia and the contraction of tubal muscle^27^,^28^, which has been considered as one of the main factors contributing to embryo retention and implantation within the fallopian tube^29^. Therefore, it is believed that the administration of LNG-EC increases the level of progesterone enough to affect the human fallopian tube, resulting in an increased risk for EP^27^,^30^. A clinical trial by WHO found that LNG-EC prevented 95% of unwanted pregnancies when taken within 24 h of unprotected intercourse; 85% if taken within 25–48 h; and 58% if taken within 49–72 hours^31^. Glasier *et al.* reported a pregnancy rate of 2.7% when LNG-EC was taken within 72–96 h and 3.0% when it was taken within 96–120 h^32^. However, our results show that the efficacy of LNG-EC does not drop significantly with the time elapsed since unprotected intercourse. LNG-EC cannot prevent pregnancies if taken after the level of luteinizing hormone has started to rise. Although the American Congress of Obstetricians and Gynecologists recommended that LNG-EC be administered as soon as possible after unprotected intercourse to maximize its efficacy^33^,^34^, but we believed that it will not reduce the risk of EP following its failure with time elapsed. Fertilization often occurs 12 h following ovulation, and the whole process lasts about 24 h. Thirty hours after fertilization, the transportation of the zygote through the fallopian tube starts, aided with the beating of tubal cilia and the contraction of smooth muscles^5^. If LNG-EC is taken after the luteinizing hormone level starts to rise, that is, when it is ineffective in preventing pregnancy, the plasma concentration of LNG-EC might still remain high during the time of embryo-tubal transport due to its half-life of 24 h; therefore, the chance of embryo-tubal implantation increases with declined tubal motility. Many women do not keep track of their last menstrual cycle, some get it wrong, some have irregular cycles, and the day of ovulation is also known to vary from one cycle to another^35^,^36^. Given these facts, it is difficult to understand the association between risk of EP following LNG-EC failure and the time interval from LMP to current use of LNG-EC.

The LNG-only pills were initially recommended for EC use in two doses of 0.75 mg with 12 hours apart; later, a single dose of 1.5 mg was proposed as an alternative and considered as effective as the split-dose regimen^13^. A pharmacokinetic study revealed that the serum peak level of LNG was 25 nmol/L following intake of the two-split doses of 0.75 mg, but rose to 40 nmol/L following administration of the single dose of 1.5 mg^37^. Compared to the split-dose regimen, a single dose with a higher serum peak of LNG could possibly result in a decline in tubal motility and thus increase the subsequent risk of EP^15^.

Some LNG-EC users got pregnancies because of improper use of LNG-EC, which is defined as “user failure” rather than “true” failure (as in cases where LNG-EC is used correctly)^35^,^38^. Further acts of intercourse following LNG-EC use in the same cycle are considered as one of the most important causative factors of user failure^39^. Although in our study, women who further indulged in intercourse following LNG-EC use in the same cycle were at no significant risk of EP when comparing to those who did not, but it was worth mentioning that after analyzing the contraceptive methods used for further intercourse following LNG-EC use, women who did not use any contraceptive methods and repeated use of LNG-EC were correlated with an increased risk of EP following LNG-EC failure compared to no further intercourse. We think that this is because fertilization might occur when unprotected intercourse happens in the period of delayed ovulation caused by the initial use of LNG-EC. If LNG-EC is repeatedly used after a delayed ovum has been fertilized, the risk of embryo retention and implantation within the fallopian tube might increase due to the reduced tubal motility caused by LNG-EC. In our study, more than half (55.88%) of the current LNG-EC users engaged in a further act of intercourse following LNG-EC use during the same cycle, which was far greater than the percentage of 30% reported by Von *et al.*^13^. This might explain the elevated risk of EP following LNG-EC failure in our study.

Since this was a hospital-based case-control study, a selection bias is inevitable. However, our study is a multicenter one in which five hospitals were enrolled and it covered the urban and rural area of Shanghai, so we were able to recruit women who were relatively good representatives of the general female population. Moreover, the OR of the relationship between contraceptive methods and risk of EP in case-control studies can vary according to the choice of control group^40^. Therefore, we chose both women with IUP and non-pregnant women as two groups of controls to thoroughly explore the association between the risk of EP and the use of LNG-EC. Another strength of this study is the large sample size that had a statistical power of 90%.

In summary, LNG-EC should be taken as soon as possible after unprotected intercourse to prevent unwanted pregnancies. Women should be informed to avoid further act of unprotected intercourse or repeated use of LNG-EC for further intercourse in the same cycle. Women and health care professional should be aware of the possible EP if LNG-EC is taken during the cycle of conception. A better knowledge on the risk of EP following LNG-EC failure holds the potential to optimize the use of LNG-EC and thus reduce the chance of unwanted pregnancy including EP occurred in the case of EC failure.

## Methods

### Ethics statement

This study was conducted from March 2011 through April 2013 at five hospitals (two general hospitals and three maternity hospitals) in Shanghai, China. The approval of regional ethics committee was obtained from the leading institute of International Peace Maternity and Child Health Hospital as well as another four institutes. All of these patients have been informed consent before collection of data and their blood samples, according to institutional guidelines. Women were informed that they had the right to refuse to participate in the study or withdraw from the study at any time, and that their information would be kept strictly confidential.

### Participant selection

The methodology of the participant selection has been described in our previous study, which was focused on studying the relationship between EP risk and common contraceptive methods used during the previous and current menstrual cycle^41^.

According to the diagnostic criteria of the American College of Obstetricians and Gynecologists Practice Bulletin^42^, women of reproductive age ranging from 17 to 45 years with diagnosed EP in the inpatient department of gynecology of each hospital were interviewed as potential subjects of EP group. Women with a history of venous thromboembolic disease, cardiovascular disease, epilepsy, cancer or any other disease that could have influenced the choice of contraceptive method were excluded. The final number of eligible EP subjects was 2559.

Two control groups were included IUP group and non-pregnancy group. Potential IUP subjects were collected from the prenatal clinic and family planning clinic of the same hospital, and potential non-pregnancy subjects were originated from the physical examination center of the hospital. The eligibility criteria for both groups were at reproductive age ranging from 17 to 45 years, without a history of vascular disease, epilepsy, cancer or any other disease that could have influenced the choice of contraceptive method. Women who used mifepristone for EC (this drug is legal in China) in the current conception/menstruation cycle were excluded because the conditions were beyond the scope of the study. Within the same hospital, IUP women were matched in terms of age (±5 years), marital status (married or unmarried) and gestational age (±7 days) at a ratio of 1:1, and the non-pregnant women were matched in terms of age (±5 years) and marital status (married or unmarried). Finally, 2534 and 2549 women were eligible for the IUP and non-pregnant control groups respectively.

### Definitions of previous contraceptive user and current contraceptive user

**Figure 2:**
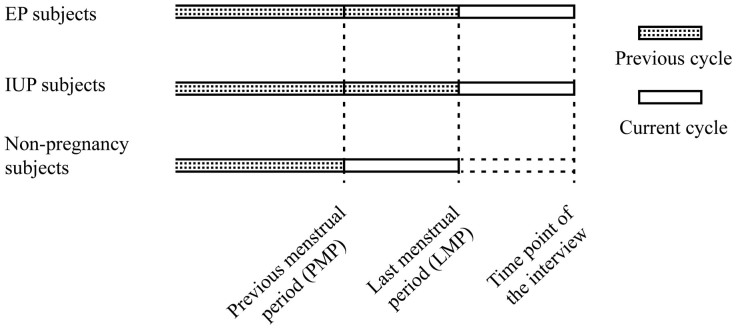
The definition of previous cycle and current cycle.

The definitions of previous contraceptive user and current contraceptive user have been described previously^41^. Previous cycle is defined as the menstrual cycle before the LMP for EP and IUP women, and the one before the previous menstrual period (PMP) for non-pregnant women. Current cycle refers to the menstrual cycle after LMP for EP and IUP women, and the one between PMP and LMP for non-pregnant women (Figure 2). A woman who had used a long-term contraceptive method including IUDs or OCPs for at least one menstrual cycle or who had used a short-term contraceptive method including ECPs or condoms at least once was considered as a user of a given method. “Previous user of contraceptives” was defined as a woman who had used any contraceptive in the previous cycle, and “current user of contraceptive” was defined as a woman who used a given contraceptive method in the current cycle. A woman who had undergone tubal sterilization was considered as a current user, and a woman who had undergone reversal of tubal sterilization was considered as a previous user. A woman was considered as a previous IUDs user if she had inserted an IUDs but later removed it, while a current user referred to a woman who was using an IUDs during the interview. Current non-users of contraception referred to women who did not use any contraceptive methods in the current cycle.

### Data and sample collection

An interview method was used, where the focus was the use of LNG-EC in two parts, previous use of LNG-EC and current use of LNG-EC.

Previous use of LNG-EC: previous use (no or yes), total number of times LNG-EC was used (not used, 1–2 times, 3–4 times or more than 5 times), number of times LNG-EC was used in the past year (not used, 0 times, 1–2 times, 3–4 times or more than 5 times), time interval between last LNG-EC use and current conception cycle (more than 12 months, 7–12 months, 4–6 months or 1–3 months).

Current use of LNG-EC: current use (yes or no), time interval between coitus and current use of LNG-EC (within 24 h, 25–48 h, 49–72 h, 73–120 h or more than 120 h), time interval between LMP and current use of LNG-EC (within 12 days, 13–14 days, 15–16 days or more than 17 days), LNG-EC regime (double doses of 0.75 mg 12 h apart or a single dose of 1.5 mg), intercourse after current use of LNG-EC (yes or no), and choice of contraceptive method for further acts of intercourse (no contraceptives, condom, repeated use of LNG-EC or other methods).

Additionally, participants were also interviewed using a structured questionnaire to obtain information about their socio-demographic characteristics, reproductive and gynecological history, previous contraceptive experience and choice of contraceptives in the current cycle.

Blood samples were also collected from each participant and tested for serum CT IgG antibody by applying enzyme-linked immunosorbent assay (ELISA; Beijing Biosynthesis Biotechnology, China) according to the manufacturer's instructions.

### Statistical analysis

Univariate χ^2^ tests were conducted to detect differences among groups with regard to socio-demographic characteristics, history of reproduction and gynecological disease, and previous and current use of contraception. The ORs and their 95% confidence intervals were calculated to estimate the association between the risk of EP and previous or current use of LNG-EC. We adjusted ORs and their 95% CIs for potential confounding factors in a mixed effects model by using institutions as a random effect.

During multivariate analyses of the association between previous use of LNG-EC and current EP occurrence, we adjusted the ORs and their 95% CIs for the following potential confounding factors: birth place (Shanghai or outside Shanghai), education attainment (collage or above, high school, middle school, or primary school or lower), occupation (employed, self-employed, or unemployed), parity (0, 1 or ≥2), previous EP (no or yes), serum CT IgG antibody test (negative or positive), previous infertility (no or yes), previous adnexal surgery (no or yes), previous appendectomy (no or yes), and previous contraceptive methods including IUDs, and other methods (no or yes), current contraceptive methods (no, IUDs, OCPs, sterilization, LNG-EC, and other methods; data was shown in our previous study^41^).

When analyzing the association between current use of LNG-EC and the risk of current EP, ORs and their 95% CIs were adjusted for all the confounding factors including birth place (Shanghai or outside Shanghai), education attainment (collage or above, high school, middle school, or primary school or lower), occupation (employed, self-employed, or unemployed), parity (0, 1 or ≥2), previous EP (no or yes), serum CT IgG antibody test (negative or positive), previous infertility (no or yes), previous adnexal surgery (no or yes), previous appendectomy (no or yes), and previous use of LNG-EC (no or yes).

We also performed tests for trend by entering categorical variables as continuous variables in the mixed effects model in order to detect their trend associations with the occurrence of EP.

All statistical analyses were performed using the SAS software, version 9.2 (SAS Institute, Inc, Cary, NC). All *p*-values were calculated using two-sided tests and differences were considered significant if the *p*-value was less than 0.05. This study had a statistical power of 90% for identifying the association between the risk of EP and previous or current use of LNG-EC. In addition, we would also like to note that in the description of this study, the ORs in the analysis between EP group and non-pregnant group were subscripted with 1, while the ORs in the analysis between EP group and IUP group were subscripted with 2.

## Author Contributions

Experimental design by H.F.H., L.C. and J.Z. Data collected by W.H.Z., X.X., S.J.C., H.P. and G.J.Q., J.Z. and C.L. analysed the data. J.Z. and C.L. wrote the manuscript. All authors discussed the results and provided comments on the manuscript.

## Figures and Tables

**Table 1:** Baseline characteristics

	EP		IUP		NonP		
	n a	(%)	n a	(%)	n a	(%)	P
**Socio-demographic characteristics**							
**Institutions **b							
1	1,404	(58.23)	1,409	(58.32)	1,408	(58.21)	1.00
2	272	(11.28)	272	(11.26)	276	(11.41)	
3	276	(11.45)	274	(11.34)	276	(11.41)	
4	291	(12.07)	293	(12.13)	293	(12.11)	
5	168	(6.97)	168	(6.95)	166	(6.86)	
**Age (year)**							
≤20	24	(1.00)	32	(1.32)	22	(0.91)	0.16
20–24	363	(15.21)	398	(16.47)	394	(16.29)	
25–29	753	(18.41)	772	(31.95)	718	(29.68)	
30–34	793	(21.55)	755	(31.25)	749	(30.96)	
35–39	332	(11.50)	322	(13.33)	368	(15.21)	
≥40	146	(4.49)	137	(5.67)	168	(6.95)	
**Birth place**							
Shanghai	698	(28.95)	775	(32.08)	927	(38.32)	<0.01
Outside of Shanghai	1,713	(71.05)	1,641	(67.92)	1,492	(61.68)	
**Marital status**							
Married	2,067	(85.80)	2,088	(86.42)	2,101	(86.85)	0.56
Unmarried	342	(15.20)	328	(13.58)	318	(13.15)	
**Education attainment**							
Collage or above	1,061	(44.01)	1,378	(57.04)	1,256	(51.92)	<0.01
High school	314	(13.02)	280	(11.59)	275	(11.37)	
Middle school	178	(7.38)	195	(8.07)	188	(7.77)	
Primary school or lower	858	(35.59)	563	(23.30)	700	(28.94)	
**Occupation**							
Employed	1,682	(69.88)	1,897	(78.58)	1,928	(79.70)	<0.01
Self-employed	262	(10.88)	184	(7.62)	209	(8.64)	
Unemployed	463	(19.24)	333	(13.79)	282	(11.66)	
**Smoking **c							
None smoker	2,298	(95.31)	2,294	(96.18)	2,290	(96.10)	0.41
Occasional smoker	63	(2.61)	57	(2.39)	57	(2.39)	
Regular smoker	50	(2.07)	34	(1.43)	36	(1.51)	
**Reproductive history**							
**Parity**							
0	1,143	(48.29)	1,280	(52.98)	639	(26.54)	<0.01
1	994	(41.99)	973	(40.27)	1,465	(60.84)	
≥2	230	(9.72)	163	(6.75)	304	(12.62)	
**Number of previous abortions**							
0	873	(36.88)	930	(38.49)	948	(39.34)	0.71
1	763	(32.23)	756	(31.29)	749	(31.08)	
2	485	(20.49)	497	(20.57)	484	(20.08)	
≥3	246	(10.39)	233	(9.64)	229	(9.50)	
**Gynecologic history**							
**Chlamydia trachomatis IgG test**							
Negative	1,648	(69.13)	2,099	(89.55)	2,181	(90.91)	<0.01
Positive	736	(30.87)	245	(10.45)	218	(9.09)	
**Previous ectopic pregnancy**							
No	2,093	(86.81)	2,363	(97.81)	2,356	(97.40)	<0.01
Yes	318	(13.19)	53	(2.19)	63	(2.60)	
**Previous infertility**							
No	2,005	(83.26)	2,286	(95.65)	2,325	(96.47)	<0.01
Yes	403	(16.74)	104	(4.35)	76	(3.15)	
**Surgical history**							
**Previous cesarean section **d							
No	691	(56.09)	624	(54.74)	988	(55.72)	0.79
Yes	541	(43.91)	516	(45.26)	785	(44.28)	
**Previous adnexal surgery**							
No	1,985	(82.33)	2,322	(96.19)	2,242	(92.84)	<0.01
Yes	426	(17.67)	92	(3.81)	173	(7.16)	
**Previous appendectomy**							
No	2,303	(95.84)	2,346	(97.47)	2,331	(96.44)	0.01
Yes	100	(4.16)	61	(2.53)	86	(3.56)	
**Previous contraceptive experience**							
**Previous use of intrauterine device**							
No	1,762	(81.99)	2,106	(88.26)	1,640	(84.32)	<0.01
Yes	387	(18.01)	280	(11.74)	305	(15.68)	
**Previous of oral contraceptive pills**							
No	2,254	(94.47)	2,295	(95.55)	2,271	(94.08)	0.06
Yes	132	(5.53)	107	(4.45)	143	(5.92)	
**Previous use of LNG-EC**							
No	1,283	(53.57)	1,311	(54.74)	1,330	(55.12)	0.53
Yes	1,112	(46.43)	1,084	(45.26)	1,083	(44.88)	
**Previous use of other contraceptive methods **e							
No	829	(35.32)	421	(17.50)	419	(17.49)	<0.01
Yes	1,518	(64.68)	1,985	(82.50)	1,976	(82.51)	

EP, ectopic pregnancy; IUP, intrauterine pregnancy; NonP, non-pregnancy; LNG-EC, levonorgestrel emergency contraception.

^a^The sum does not necessarily equal the sample size for all variables because of missing data.

^b^Center 1 = International Peace Maternity and Child Health Hospital; Center 2 = Shanghai First People's Hospital; Center 3 = Songjiang Central Hospital; Center 4 = Songjiang Maternity and Child Health Hospital; Center 5 = Minhang Central Hospital.

^c^Occasional smoker: cigarette smoking more than 4 times a week, but a day on average less than one cigarette. Regular smoker: smoking more than one cigarettes per day, continuously or over a period of 6 months.

^d^The number of women who had delivered a child was used as the denominator to calculate the percentage.

^e^Other contraceptive methods includes condom use, rhythm method, and withdrawal.

**Table 2:** Previous use of LNG-EC

	EP		IUP		NonP		OR_1_ [95%CI]	OR_2_ [95%CI]	AOR_1_ [95%CI]^b^	AOR_2_ [95%CI]^b^
	n a	(%)	n a	(%)	n a	(%)	EP vs. NonP	EP vs. IUP	EP vs. NonP	EP vs. IUP
**Previous use of LNG-EC**										
Not used	1,283	(53.57)	1,311	(54.74)	1,330	(55.12)	*Reference*	*Reference*	*Reference*	*Reference*
Yes	1,112	(46.43)	1,084	(45.26)	1,083	(44.88)	0.94 [0.84, 1.05]	0.95 [0.85, 1.07]	0.99 [0.85, 1.18]	0.95 [0.82, 1.10]
**Total times of LNG-EC use**										
Not used	1,283	(53.57)	1,311	(54.74)	1,330	(55.12)	*Reference*	*Reference*	*Reference*	*Reference*
1–2	585	(24.43)	568	(23.72)	566	(23.46)	1.07 [0.93, 1.23]	1.05 [0.92, 1.21]	1.08 [0.89, 1.31]	1.17 [0.99, 1.38]
3–4	290	(12.11)	305	(12.73)	334	(13.84)	0.90 [0.75, 1.07]	0.97 [0.81, 1.16]	0.89 [0.69, 1.13]	0.93 [0.74, 1.17]
≥5	237	(9.89)	211	(8.81)	183	(7.58)	1.34 [1.09, 1.65]	1.15 [0.94, 1.40]	0.94 [0.70, 1.26]	0.85 [0.66, 1.11]
*P for trend*							0.15	0.35	0.71	0.67
**Times of LNG-EC use in the past one year**										
Not used	1,283	(53.57)	1,311	(54.74)	1,330	(55.12)	*Reference*	*Reference*	*Reference*	*Reference*
0	542	(22.63)	566	(23.63)	659	(27.31)	0.85 [0.74, 0.98]	0.98 [0.85, 1.13]	0.94 [0.78, 1.13]	1.15 [0.97, 1.36]
1–2	349	(14.57)	355	(14.82)	294	(12.18)	1.23 [1.04, 1.46]	0.96 [0.82, 1.14]	1.08 [0.84, 1.39]	0.89 [0.71, 1.10]
3–4	125	(5.22)	99	(4.13)	74	(3.07)	1.75 [1.30, 2.36]	1.52 [1.14, 2.03]	1.16 [0.78, 1.72]	1.05 [0.74, 1.50]
≥5	96	(4.01)	64	(2.67)	56	(2.32)	1.78 [1.27, 2.49]	1.53 [1.11, 2.12]	1.31 [0.83, 2.06]	0.92 [0.61, 1.39]
*P for trend*							<10^−4^	0.01	0.47	0.75
**Last use-current cycle interval (months)**										
Not used	1,283	(53.57)	1,311	(54.74)	1,330	(55.12)	*Reference*	*Reference*	*Reference*	*Reference*
>12	542	(22.63)	566	(23.63)	659	(27.31)	0.85 [0.74, 0.98]	0.98 [0.85, 1.13]	0.94 [0.78, 1.13]	1.15 [0.97, 1.36]
7–12	231	(9.65)	246	(10.27)	199	(8.24)	1.20 [0.98, 1.48]	0.96 [0.79, 1.17]	1.13 [0.85, 1.50]	0.88 [0.68, 1.13]
4–6	189	(7.89)	173	(7.22)	150	(6.22)	1.31 [1.04, 1.64]	1.12 [0.90, 1.39]	1.04 [0.75, 1.44]	0.85 [0.64, 1.13]
1–3	150	(6.26)	99	(4.13)	75	(3.11)	2.07 [1.56, 2.76]	1.55 [1.19, 2.02]	1.26 [0.86, 1.85]	1.13 [0.81, 1.57]
*P for trend*							<10^−4^	0.01	0.37	0.86

EP, ectopic pregnancy; IUP, intrauterine pregnancy; NonP, non-pregnancy; LNG-EC, levonorgestrel emergency contraception; OR, odds ratio; AOR, adjusted odds ratio; CI, confidence interval.

^a^The sum does not necessarily equal the sample size for all variables because of missing data.

^b^Institutions was used as a random effect in the mixed effects model to adjusted ORs for birth place, education attainment, occupation, parity, previous ectopic pregnancy, presence of serum Chlamydia trachomatis IgG, previous infertility, previous adnexal surgery, previous appendectomy, previous contraceptive methods (IUDs and other contraceptive methods), and current contraceptive methods (no, IUDs, OCPs, sterilization, LNG-EC, and other contraceptive methods).

**Table 3:** Current use of LNG-EC

	EP		IUP		NonP		OR_1_ [95%CI]	OR_2_ [95%CI]	AOR_1_ [95%CI] b	AOR_2_ [95%CI] b
	n a	(%)	n a	(%)	n a	(%)	EP vs. NonP	EP vs. IUP	EP vs. NonP	EP vs. IUP
**Current contraceptive methods**										
Non-users	1,337	(55.87)	1,585	(65.77)	272	(11.33)	*Reference*	*Reference*	*Reference*	*Reference*
None LNG-EC methods	715	(29.88)	680	(28.22)	2,032	(84.67)	0.07 [0.06, 0.08]	1.25 [1.10, 1.42]	0.10 [0.08, 0.12]	1.79 [1.52, 2.11]
LNG-EC	341	(14.25)	145	(6.02)	96	(4.00)	0.72 [0.56, 0.94]	2.79 [2.27, 3.43]	1.04 [0.76, 1.42]	5.29 [4.07, 6.87]
**Coitus-administration interval (hours)**										
Non-users	1,337	(79.68)	1,585	(91.62)	272	(74.73)	*Reference*	*Reference*	*Reference*	*Reference*
<24	185	(11.03)	83	(4.80)	54	(14.84)	0.70 [0.50, 0.97]	2.64 [2.02, 3.46]	0.78 [0.52, 1.17]	4.55 [3.25, 6.38]
25–48	96	(5.72)	38	(2.20)	25	(6.87)	0.78 [0.49, 1.24]	2.30 [2.04, 4.39]	0.90 [0.52, 1.54]	5.26 [3.36, 8.25]
49–72	37	(2.21)	16	(0.92)	9	(2.47)	0.84 [0.40, 1.76]	2.74 [1.52, 4.95]	0.99 [0.45, 2.18]	5.10 [2.64, 9.84]
73–120	23	(1.37)	8	(0.46)	4	(1.10)	1.17 [0.40, 3.42]	3.41 [1.52, 7.64]	1.92 [0.55, 6.74]	4.63 [1.96, 10.95]
*P for trend*							0.22	<0.01	0.28	0.74
**LMP-administration interval (days)**										
Non-users	1,337	(79.68)	1,585	(91.62)	272	(74.73)	*Reference*	*Reference*	*Reference*	*Reference*
≤12	78	(4.65)	32	(1.85)	20	(5.49)	0.80 [0.48, 1.32]	2.89 [1.90, 4.39]	0.94 [0.52, 1.68]	4.57 [2.82, 7.42]
13–14	68	(4.05)	34	(1.97)	20	(5.49)	0.69 [0.41, 1.16]	2.37 [1.56, 3.60]	0.79 [0.44, 1.41]	4.28 [2.64, 6.95]
15–16	45	(2.68)	19	(1.10)	13	(3.57)	0.71 [0.38, 1.33]	2.81 [1.63, 4.82]	0.76 [0.38, 1.53]	3.91 [2.14, 7.14]
≥17	150	(8.94)	60	(3.47)	39	(10.71)	0.78 [0.54, 1.14]	2.96 [2.18, 4.03]	0.93 [0.59, 1.46]	5.65 [3.86, 8.27]
**Way of taking LNG-EC**										
Non-users	1,337	(79.68)	1,585	(91.62)	272	(74.73)	*Reference*	*Reference*	*Reference*	*Reference*
Double doses of 0.75 mg 12 h apart	37	(2.21)	26	(1.50)	24	(6.59)	0.31 [0.19, 0.53]	1.69 [1.02, 2.80]	0.36 [0.20, 0.67]	3.14 [1.73, 5.71]
A single dose of 1.5 mg	304	(18.12)	119	(6.88)	68	(18.68)	0.91 [0.68, 1.22]	3.03 [2.42, 3.79]	1.06 [0.73, 1.55]	5.15 [3.87, 6.86]
**Further acts of intercourse after use of LNG-EC **c										
No further intercourse	145	(8.64)	61	(3.53)	49	(13.46)	*Reference*	*Reference*	*Reference*	*Reference*
Yes	196	(11.68)	84	(4.86)	43	(11.81)	0.93 [0.65, 1.32]	2.77 [2.12, 3.61]	1.45 [0.89, 2.37]	1.15 [0.74, 1.79]
**Contraceptive methods of further acts of intercourse **c										
No further intercourse	145	(42.52)	61	(42.07)	49	(53.26)	*Reference*	*Reference*	*Reference*	*Reference*
Not used	80	(23.46)	39	(26.90)	14	(15.22)	1.93 [1.00, 3.71]	0.86 [0.53, 1.40]	2.35 [1.17, 4.71]	1.04 [0.60, 1.81]
Condom	54	(15.84)	30	(20.69)	21	(22.83)	0.87 [0.48, 1.58]	0.76 [0.44, 1.30]	0.98 [0.51, 1.93]	0.79 [0.43, 1.45]
Repeated use of LNG-EC	47	(13.78)	7	(4.83)	5	(5.43)	3.18 [1.20, 8.44]	2.83 [1.21, 6.60]	3.08 [1.09, 8.71]	2.49 [1.00, 6.19]
Other d	15	(4.40)	8	(5.52)	3	(3.26)	1.69 [0.47, 6.08]	0.79 [0.32, 1.96]	2.01 [0.53, 7.67]	0.69 [0.25, 1.89]

EP, ectopic pregnancy; IUP, intrauterine pregnancy; NonP, non-pregnancy; LNG-EC, levonorgestrel emergency contraception; OR, odds ratio; AOR, adjusted odds ratio; CI, confidence interval.

^a^The sum does not necessarily equal the sample size for all variables because of missing data.

^b^Institutions was used as a random effect in the mixed effects model to adjusted ORs for confounding factors including birth place, education attainment, occupation, parity, previous ectopic pregnancy, presence of serum Chlamydia trachomatis IgG, previous infertility, previous adnexal surgery, previous appendectomy, previous use of LNG-EC.

^c^The number of women who had repeated intercourse after LNG-EC use in the same cycle was used as the denominator to calculate the percentage. ORs were calculated by using multivariate logistic regression with adjustment for confounding factors described above as well as institutions, because the mixed effects model cannot be applied in these variables.

^d^Other contraceptive methods includes rhythm method, and withdrawal.
